# Supplementary material for: BASIN: Bayesian mAtrix variate normal model with Spatial and sparsIty priors in Non-negative deconvolution
Source: ArXiv. 2025 Oct 24:arXiv:2510.16130v2. Preprint. [Version 2] (PMC12633627)
Supplement: Supplement 1 [file NIHPP2510.16130v2-supplement-1.pdf]

Supplementary Information

1 Supplementary figures

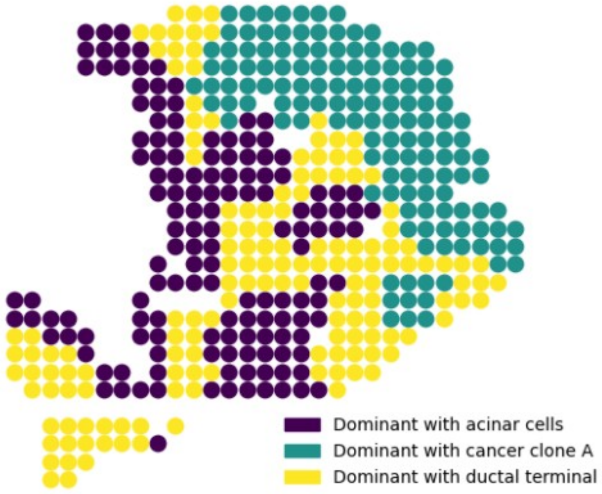

Figure 1: Spatial division of our simulated data. The simulation is based on the real PDAC-A data [35]. We divide the spatial domain into three regions according to the histological annotation, and assume there are three cell types (acinar, cancer cluster A and terminal ductal cells) whose proportions follow different probability distributions and are dominant respectively in the three regions.

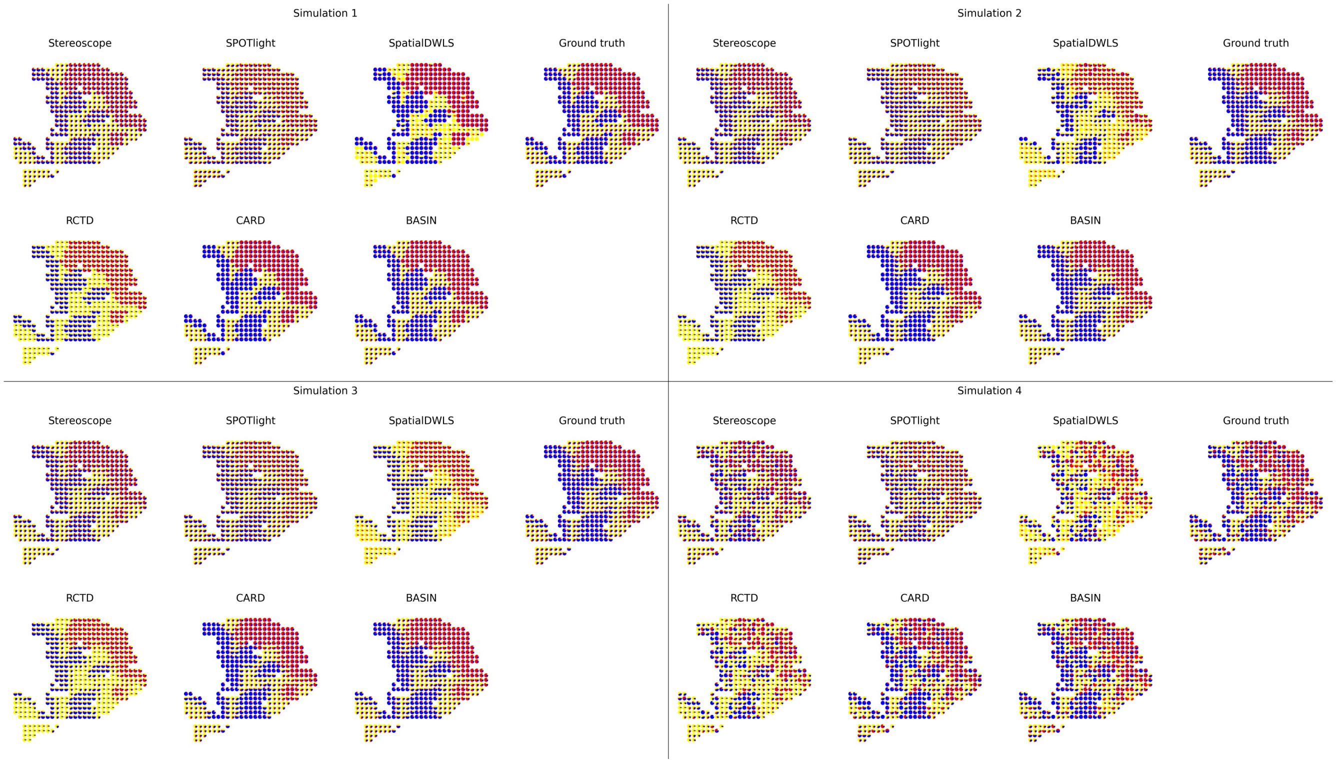

Figure 2: The results of the four simulation studies compared with the ground truth. Blue: acinar cells. Yellow: terminal ductal cells. Red: cancer cells.

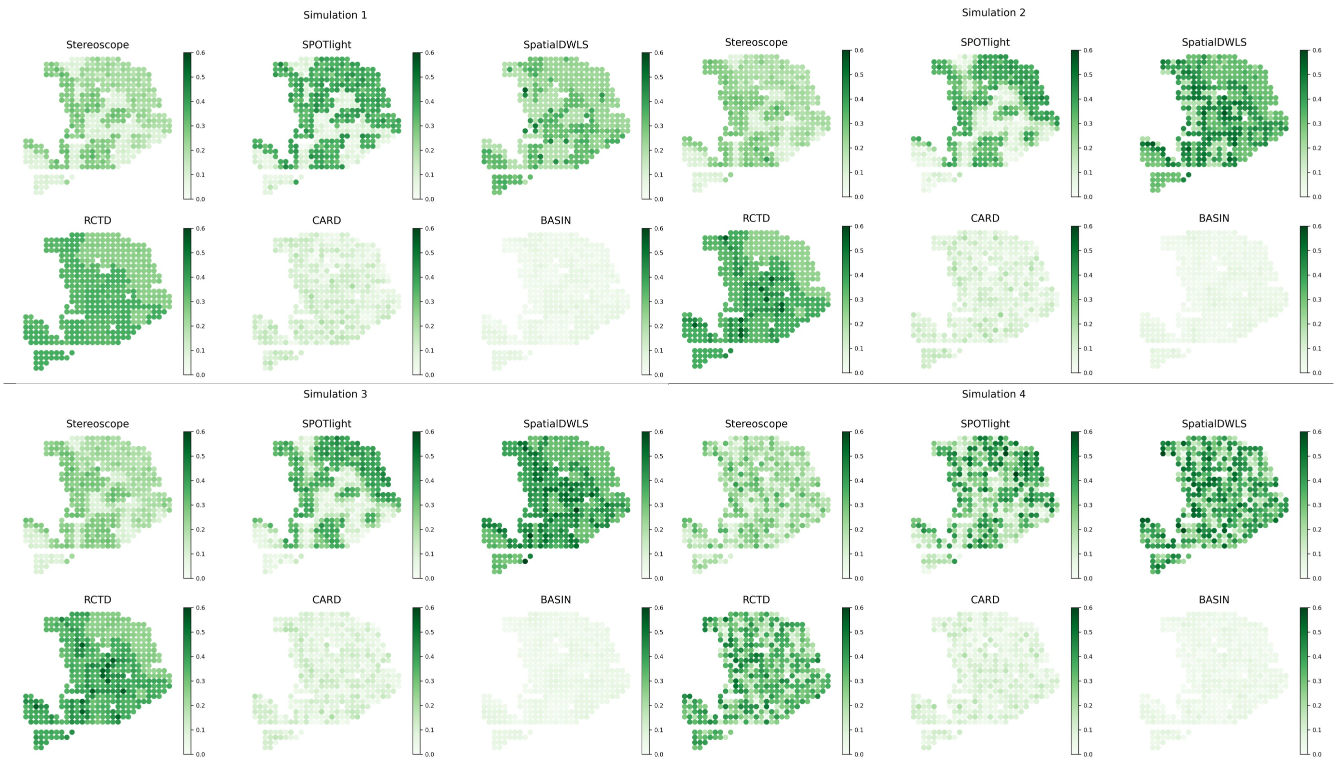

Figure 3: The spot-wise RMSE of the four simulations between the six compared methods and the ground truth.

2 Details of the method

2.1 Matrix normal distribution

Matrix normal distribution is a generalization of multivariate normal distribution. Suppose there is a random matrix $\mathbf{X} \in \mathbb{R}^{n\times p}$ whose entries follow normal distributions, and the mean values of $\mathbf{X}$ is a matrix $\mathbf{M}$ of size $n \times p$. Each column of $\mathbf{X}$ follows a multivariate normal distribution with covariance matrix $\mathbf{U}$ of size $n \times n$. Each row of $\mathbf{X}$ follows a multivariate normal distribution with covariance matrix $\mathbf{V}$ of size $p \times p$. Then $\mathbf{X}$ follows a matrix normal distribution $\mathbf{X} \sim \mathcal{MN}(\mathbf{M}, \mathbf{U}, \mathbf{V})$ where $\mathbf{U}$ and $\mathbf{V}$ are column and row covariances. Its probability density function is

$$P(\mathbf{X}) = \frac{\exp(-\frac{1}{2}\mathbf{tr}(\mathbf{V}^{-1}(\mathbf{X}-\mathbf{M})^T\mathbf{U}^{-1}(\mathbf{X}-\mathbf{M}))}{(2\pi)^{np/2}\det(\mathbf{U})^{p/2}\det(\mathbf{V})^{n/2}} \tag{1}$$

On the other hand, if the probability density function of a random matrix $\mathbf{X} \in \mathbb{R}^{n\times p}$ satisfies

$$P(\mathbf{X}) \propto \exp(-\frac{1}{2}\mathbf{tr}(\mathbf{A}\mathbf{X}^T\mathbf{B}\mathbf{X} - \mathbf{2}\mathbf{C}\mathbf{X})) \tag{2}$$

where $\mathbf{A}$ and $\mathbf{B}$ are symmetric, then $\mathbf{X}$ can be represented in form of matrix normal distribution:

$$\begin{aligned}
P(\mathbf{X}) &\propto \exp(-\frac{1}{2}\mathbf{tr}(\mathbf{A}\mathbf{X}^T\mathbf{B}\mathbf{X} - \mathbf{2}\mathbf{C}\mathbf{X})) \\
&\propto \exp(-\frac{1}{2}\mathbf{tr}(\mathbf{A}\mathbf{X}^T\mathbf{B}\mathbf{X} - \mathbf{2}\mathbf{A}\mathbf{A}^{-1}\mathbf{C}\mathbf{B}^{-1}\mathbf{B}\mathbf{X})) \\
&\propto \exp(-\frac{1}{2}\mathbf{tr}(\mathbf{A}\mathbf{X}^T\mathbf{B}\mathbf{X} - \mathbf{2}\mathbf{A}\mathbf{A}^{-1}\mathbf{C}\mathbf{B}^{-1}\mathbf{B}\mathbf{X} + \mathbf{A}(\mathbf{A}^{-1}\mathbf{C}\mathbf{B}^{-1})\mathbf{B}(\mathbf{B}^{-T}\mathbf{C}^T\mathbf{A}^{-T}))) \\
&\propto \exp(-\frac{1}{2}\mathbf{tr}(\mathbf{A}(\mathbf{X} - (\mathbf{B}^{-T}\mathbf{C}^T\mathbf{A}^{-T})^T\mathbf{B}(\mathbf{X} - (\mathbf{B}^{-T}\mathbf{C}^T\mathbf{A}^{-T}))
\end{aligned}$$

which is equivalent to the pdf of the matrix normal distribution

$$\mathcal{MN}(\mathbf{B}^{-T}\mathbf{C}^T\mathbf{A}^{-T}, \mathbf{B}^{-1}, \mathbf{A}^{-1}) \tag{3}$$

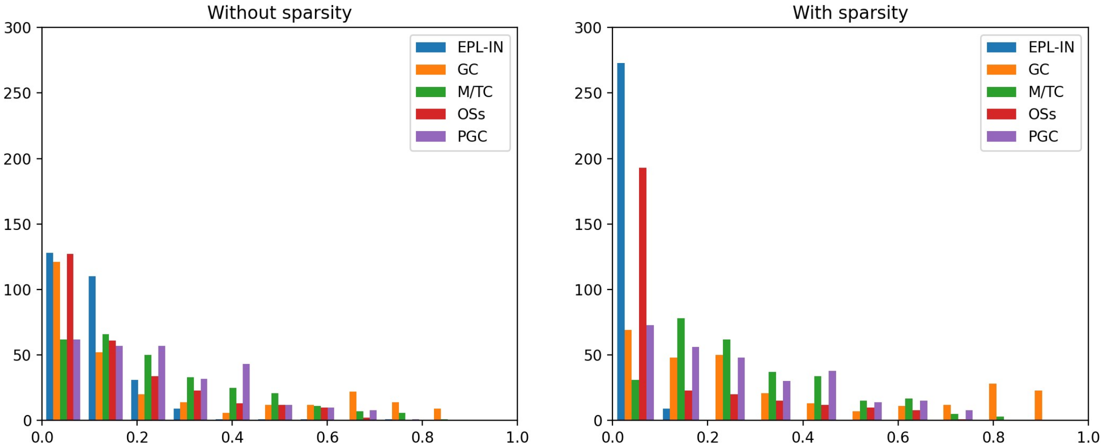

Figure 4: We make an experiment to show the effect of the exponential distribution in the prior of V. It not only constrains the values of V to be nonnegative, but also introduce sparsity to V to reduce noise and better distinguish the cells. We use the MOB data as an example and plot the histograms of the five cell types without (left) and with (right) the exponential distribution in the prior. We can see that by introducing sparsity, there are more spots with trivial proportion of EPL-IN (blue) and OSs (red) on the right. It matches the fact that EPL-IN is trivial in the tissue and OSs only exist in the a small area. On the other hand, there are more spots with high proportions of GC cells (orange) on the right, which is also reasonable since GC cells mainly distributed in the central dominant area. Therefore, the exponential distribution in the prior help us better identify the dominant cell type of each spot, and avoid getting too uniform distributed results.

2.2 Posterior of V

In the main text we have defined the following likelihood and priors

$$\mathbf{X}|\mathbf{V},\sigma^2 \sim \mathcal{MN}(\mathbf{BV},\sigma^2\mathbf{I_n},\mathbf{L}^{-1}) \tag{4}$$

$$\mathbf{V}_{ij}|\eta \sim \mathrm{Exp}(\eta) \qquad 1 \le i \le c \quad 1 \le j \le p \tag{5}$$

Their probability density function (pdf) of likelihood satisfies

$$\begin{aligned} P(\mathbf{X}|\mathbf{V},\sigma^2) &\propto \frac{1}{|\sigma^2|^{np/2}}\exp(-\frac{1}{2\sigma^2}\mathbf{tr}(\mathbf{L}(\mathbf{X}-\mathbf{BV})^T(\mathbf{X}-\mathbf{BV}))) \\ &\propto \exp(-\frac{1}{2\sigma^2}\mathbf{tr}(\mathbf{L}(\mathbf{X}-\mathbf{BV})^T(\mathbf{X}-\mathbf{BV}))) \\ &\propto \exp(-\frac{1}{2\sigma^2}\mathbf{tr}(\mathbf{LV}^T\mathbf{B}^T\mathbf{BV}-\mathbf{2LX}^T\mathbf{BV}+\mathbf{LX}^T\mathbf{X})) \\ &\propto \exp(-\frac{1}{2\sigma^2}\mathbf{tr}(\mathbf{LV}^T\mathbf{B}^T\mathbf{BV}-\mathbf{2LX}^T\mathbf{BV})) \end{aligned} \tag{6}$$

The pdf of $\mathbf{V}_{ij}|\eta$ satisfies

$$P(\mathbf{V}_{ij}|\eta) = \eta\exp(-\eta\mathbf{V}_{ij}) \quad \mathbf{V}_{ij} \ge 0 \tag{7}$$

$$P(\mathbf{V}|\eta) = \prod_{i,j} P(\mathbf{V}_{ij}|\eta) = \eta^{cp}\exp(-\eta\sum\mathbf{V}_{ij}) = \eta^{cp}\exp(-\eta\mathbf{tr}(\mathbf{J_V}^T\mathbf{V})) \tag{8}$$

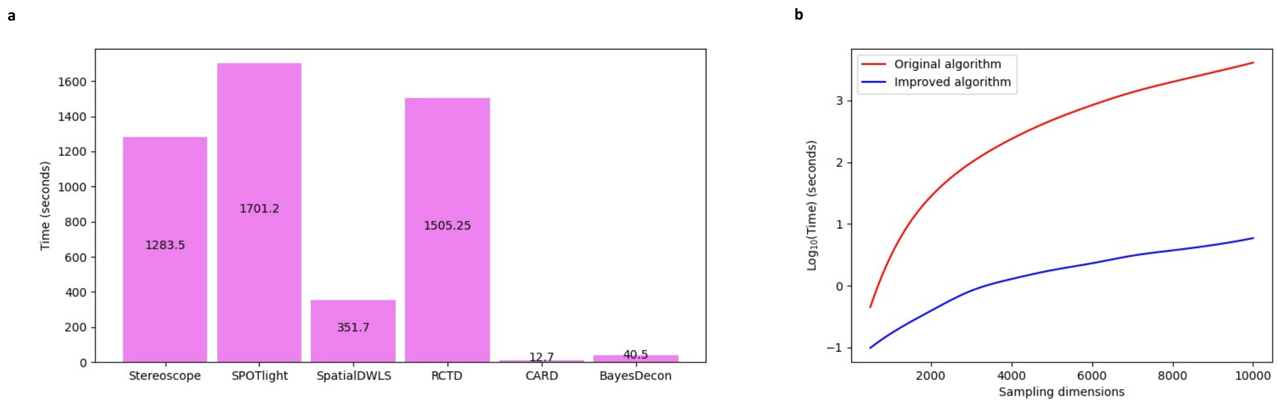

Figure 5: Computational efficiency. **a**, We use the PDAC-A data to compare the computational efficiency of the six compared methods. All the methods are implemented with 11th Gen Intel(R) Core(TM) i7-11800H @ 2.30GHz and Stereoscope also utilizes NVIDIA GeForce RTX 3080 Laptop GPU. We run Stereoscope for 5000 epochs for both the spatial transcriptomics and scRNA-seq data, and the time of BASIN means that for one sample. The other four methods are implemented in the default settings. **b**, The times of generating one sample from a truncated multivariate normal distribution vs sampling dimensions. Red: the algorithm in [41]. Blue: our improved algorithm. In practice we generate one sample by ignoring first five samples and take the sixth sample.

where $\mathbf{J_V}$ is an "all ones matrix" of the same size as $\mathbf{V}$. According to Bayes's formula,

$$\begin{aligned} P(\mathbf{V}|\mathbf{X}) &\propto P(\mathbf{X}|\mathbf{V})P(\mathbf{V}) \\ &\propto \exp(-\frac{1}{2\sigma^2}\mathbf{tr}(\mathbf{L}\mathbf{V}^T\mathbf{B}^T\mathbf{B}\mathbf{V} - \mathbf{2}\mathbf{L}\mathbf{X}^T\mathbf{B}\mathbf{V}))\exp(-\eta\mathbf{tr}(\mathbf{J_V}^T\mathbf{V})) \\ &\propto \exp(-\frac{1}{2\sigma^2}\mathbf{tr}(\mathbf{L}\mathbf{V}^T\mathbf{B}^T\mathbf{B}\mathbf{V} - \mathbf{2}\mathbf{L}\mathbf{X}^T\mathbf{B}\mathbf{V} + 2\sigma^2\eta\mathbf{J_V}^T\mathbf{V})) \\ &\propto \exp(-\frac{1}{2\sigma^2}\mathbf{tr}(\mathbf{L}\mathbf{V}^T\mathbf{B}^T\mathbf{B}\mathbf{V} - \mathbf{2}(\mathbf{L}\mathbf{X}^T\mathbf{B} - \sigma^2\eta\mathbf{J_V}^T)\mathbf{V})) \end{aligned}$$

Applying the equation 3, it satisfies a matrix normal distribution with the mean matrix:

$$\begin{aligned} \mathbf{M} &= (\mathbf{B}^T\mathbf{B})^{-T}(\mathbf{L}\mathbf{X}^T\mathbf{B} - \sigma^2\eta\mathbf{J_V}^T)^T\mathbf{L}^{-T} \\ &= (\mathbf{B}^T\mathbf{B})^{-1}(\mathbf{B}^T\mathbf{X}\mathbf{L} - \sigma^2\eta\mathbf{J_V})\mathbf{L}^{-1} \\ &= (\mathbf{B}^T\mathbf{B})^{-1}(\mathbf{B}^T\mathbf{X} - \sigma^2\eta\mathbf{J_V}\mathbf{L}^{-1}) \end{aligned} \tag{9}$$

The two covariance matrices are $(\mathbf{B}^T\mathbf{B})^{-1}$ and $\mathbf{L}^{-1}$, and the variance σ^2 can be merged with either of them. Considering that the exponential distribution is constraint to be nonnegative, the posterior of $\mathbf{V}$ follows the truncated matrix normal distribution:

$$\mathbf{V}|\mathbf{X} \sim \mathcal{TMN}((\mathbf{B}^T\mathbf{B})^{-1}(\mathbf{B}^T\mathbf{X} - \sigma^2\eta\mathbf{J_V}\mathbf{L}^{-1}),\ (\mathbf{B}^T\mathbf{B})^{-1}\sigma^2,\ \mathbf{L}^{-1}) \quad (\mathbf{V} \geq 0) \tag{10}$$

2.3 Posterior of σ^2

With $\sigma^2 \sim$ Inverse-Gamma(a_2, b_2), the pdf satisfies

$$P(\sigma^2) \propto |\sigma^2|^{-a_2-1}\exp(-\frac{b_2}{\sigma^2}) \tag{11}$$

According to equation 6 we have (let $\mathbf{E} = \mathbf{X} - \mathbf{B}\mathbf{V}$)

$$P(\mathbf{X}|\sigma^2) \propto \frac{1}{|\sigma^2|^{np/2}}\exp(-\frac{1}{2\sigma^2}\mathbf{tr}(\mathbf{L}\mathbf{E}^T\mathbf{E})) \tag{12}$$

Then

$$\begin{aligned} P(\sigma^2|\mathbf{X}) &\propto P(\mathbf{X}|\sigma^2)P(\sigma^2) \\ &\propto |\sigma^2|^{-np/2-a_2-1}\exp(\frac{1}{\sigma^2}(-b_2 - \frac{\mathbf{tr}(\mathbf{L}\mathbf{E}^T\mathbf{E})}{2})) \end{aligned}$$

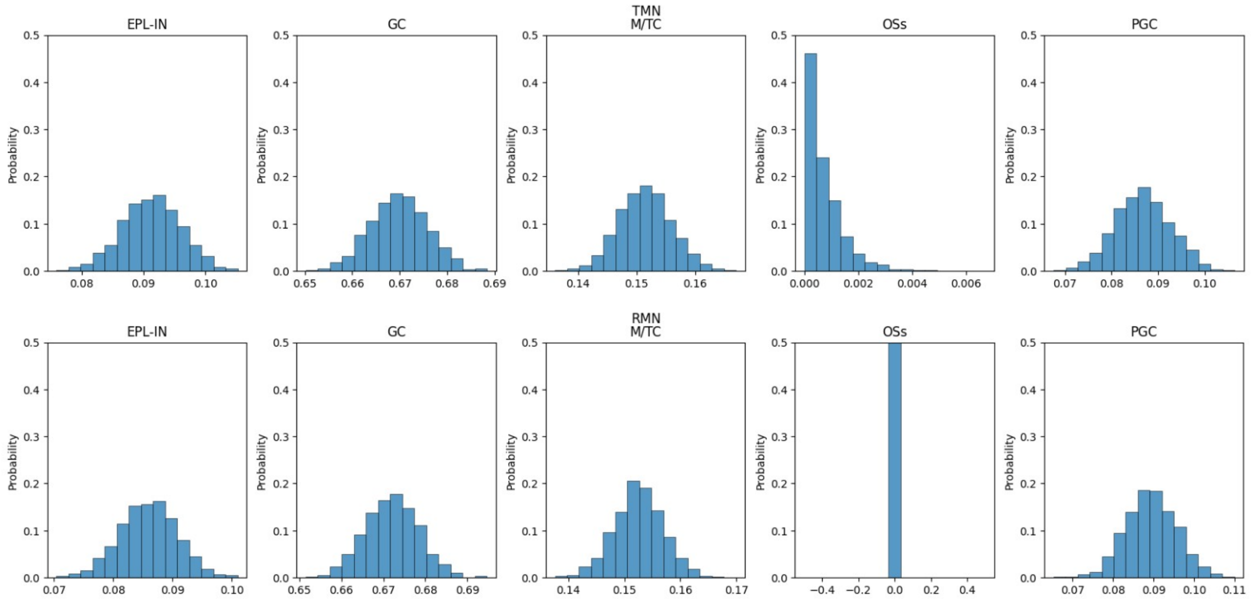

Figure 6: We make an experiment to compare rectified matrix normal (RMN) and truncated matrix normal (TMN) distribution. We use the MOB data and plot the probability histograms of the cell type proportions at the same spot as in the main text, still calculated with 2000 samples. We can observe that when the mean values are far from zero (like GC and M/TC), the samples of RMN are really close to those of TMN. When the mean values are close to zero (like EPL-IN, OSs and PGC), the difference between RMN and TMN is getting obvious. However, sampling from RMN is much faster than TMN.

So the posterior of σ^2 follows the inverse Gamma distribution

$$\sigma^2|\mathbf{X} \sim \text{Inverse-Gamma}(a_2 + np/2, \quad b_2 + \frac{\mathbf{tr}(\mathbf{L}\mathbf{E}^T\mathbf{E})}{2})) \tag{13}$$

2.4 Posterior of η

With $\eta \sim \text{Gamma}(a_3, b_3)$, the pdf satisfies

$$P(\eta) \propto \eta^{a_3-1}\exp(-\frac{\eta}{b_3}) \tag{14}$$

According to equation 8 we have

$$P(\mathbf{V}|\eta) \propto \eta^{cp}\exp(-\eta\mathbf{tr}(\mathbf{J_V}^T\mathbf{V})) \tag{15}$$

$$P(\eta|\mathbf{V}) \propto P(\mathbf{V}|\eta)P(\eta) \propto \eta^{cp+a_3-1}\exp(-\eta(\mathbf{tr}(\mathbf{J_V}^T\mathbf{V}) + \frac{1}{b_3})) \tag{16}$$

So the posterior of η follows the Gamma distribution

$$\eta|\mathbf{V} \sim \text{Gamma}(a_3 + cp, \quad 1/(\mathbf{tr}(\mathbf{J_V}^T\mathbf{V}) + \frac{1}{b_3})) \tag{17}$$

3 Sampling from nonnegative multivariate normal distribution

We sample from truncated multivariate normal distribution (TMVN) based on the method proposed in [41] in which a Gibbs sampler is built. To improve the efficiency, we only consider the case that the lower bound is zero and the upper bound is infinity. Here we briefly describe the algorithm and how we improve it. Given an n-dimensional multivariate normal distribution truncated in $[0, \infty)$:

$$\mathbf{w} \sim \mathcal{TMVN}(\mu, \mathbf{\Sigma}) \quad \mathbf{w} \geq \mathbf{0} \tag{18}$$

whose covariance $\mathbf{\Sigma}$ is positive-definite, one can find the Choleskey decomposition $\mathbf{\Sigma} = \mathbf{L}\mathbf{L}^T$ and introduce the transformation $\mathbf{x} = \mathbf{L}^{-1}(\mathbf{w} - \mu)$. The new random variable $\mathbf{x}$ follows

$$\mathbf{x} \sim \mathcal{TMVN}(\mathbf{0}, \mathbf{I}) \quad \mathbf{Lx} \geq -\mu \tag{19}$$

Then it's proved in [41] that the ith variate of $\mathbf{x}$ follows the conditional distribution:

$$\mathbf{x_i}|\mathbf{x_{-i}} \sim \mathcal{TN}(0,1) \quad \mathbf{L_i x_i} \geq -\mu - \mathbf{L_{-i} x_{-i}} \tag{20}$$

where $\mathbf{x_{-i}} = (\mathbf{x_1}, \cdots, \mathbf{x_{i-1}}, \mathbf{x_{i+1}}, \cdots, \mathbf{x_n})$ represents the $(n-1) \times 1$ vector by removing the ith entry of $\mathbf{x}$, and $\mathbf{L_{-i}}$ represents the $n \times (n-1)$ matrix by removing the ith column of $\mathbf{L}$. Sampling from the truncated univariate normal distribution in Equation 20 is not discussed here. The original algorithm can be summerized as Algorithm 1:

Algorithm 1

Given: μ, $\mathbf{\Sigma}$, sample size T, burn-in period T_b
Initialize: choose $\mathbf{x}^0$, compute $\mathbf{\Sigma} = \mathbf{L}\mathbf{L}^T$
for $t = 1$ to T **do**
  for $i = 1$ to n **do**
    Define $\mathbf{x^t_{-i}} = (\mathbf{x^t_1}, \cdots, \mathbf{x^t_{i-1}}, \mathbf{x^{t-1}_{i+1}}, \cdots, \mathbf{x^{t-1}_n})$
    Sample from $\mathbf{x^t_i}|\mathbf{x^t_{-i}} \sim \mathcal{TN}(0,1) \quad \mathbf{L_i x^t_i} \geq -\mu - \mathbf{L_{-i} x^t_{-i}}$
    Update $\mathbf{x^t} = (\mathbf{x^t_1}, \cdots, \mathbf{x^t_i}, \mathbf{x^{t-1}_{i+1}}, \cdots, \mathbf{x^{t-1}_n})$
  end for
  $\mathbf{w^t} = \mathbf{Lx^t} + \mu$
end for
Discard $\mathbf{w}^1 \cdots \mathbf{w}^{T_b}$

We improve the algorithm by replacing the matrix-vector multiplication in each step with scalar-vector multiplication. Therefore the larger the sampling dimension is, the more computation time we can save (Fig. 5b). The improved sampling algorithm is summarized in Algorithm 2:

Algorithm 2

Given: μ, $\mathbf{\Sigma}$, sample size T, burn-in period T_b
Initialize: choose $\mathbf{x}^0$, compute $\mathbf{\Sigma} = \mathbf{L}\mathbf{L}^T$, $\mathbf{z} = \mathbf{Lx^0}$
for $t = 1$ to T **do**
  for $i = 1$ to n **do**
    Define $\mathbf{x^t_{-i}} = (\mathbf{x^t_1}, \cdots, \mathbf{x^t_{i-1}}, \mathbf{x^{t-1}_{i+1}}, \cdots, \mathbf{x^{t-1}_n})$
    Sample from $\mathbf{x^t_i}|\mathbf{x^t_{-i}} \sim \mathcal{TN}(0,1) \quad \mathbf{L_i x^t_i} \geq -\mu - \mathbf{z} + \mathbf{L_i x^{t-1}_i}$
    Update $\mathbf{x^t} = (\mathbf{x^t_1}, \cdots, \mathbf{x^t_i}, \mathbf{x^{t-1}_{i+1}}, \cdots, \mathbf{x^{t-1}_n})$
    Update $\mathbf{z} = \mathbf{z} + \mathbf{L_i}(\mathbf{x^t_i} - \mathbf{x^{t-1}_i})$
  end for
  $\mathbf{w^t} = \mathbf{Lx^t} + \mu$
end for
Discard $\mathbf{x}^0 \cdots \mathbf{x}^{T_{b-1}}$

---

4 Details of datasets

Dataset	# genes	# spots	H&E image	scRNA-seq data
MOB (Replicate 12) [37]	16034	282	Yes	GSE121891 [38]
Human PDAC-A-1 (GSM3036911) [35]	19738	428	Yes	GSE111672 (PDAC-A) [35]
Human PDAC-B (GSM3405534) [35]	19738	224	No	GSE111672 (PDAC-B) [35]
Mouse brain cortex (seqFISH+) [39]	10000	524	No	GSE102827 [40]

Table 1: Spatial transcriptomics data we used in our studies.

Cell type	#
All	12801
EPL-IN (external plexiform layer interneurons)	161
GC (granule cells)	8614
MT-C (mitral and tufted cells)	1133
OSNs (olfactory sensory neurons)	1200
PGC (periglomerular cells)	1693

Table 2: Detailed cell type information of the scRNA-seq dataset GSE121891.

Cell type	#
All	48266
Astrocytes	7039
Endothelial cells	4071
Excitatory cells layer 2&3	2963
Excitatory cells layer 4	3198
Excitatory cells layer 5	3793
Excitatory cells layer 6	3276
Excitatory cells	1057
Interneurons	936
Macrophage	537
Microglia	10158
Mural	782
Oligodendrocytes	10456

Table 3: Detailed cell type information of the scRNA-seq dataset GSE102827.

Cell type	#
All	1926
Acinar cells	13
Cancer clone A	126
Cancer clone B	170
Ductal - APOL high/hypoxic	215
Ductal - CRISP3 high/centroacinar like	529
Ductal - MHC Class II	287
Ductal - terminal ductal like	350
Endocrine cells	3
Endothelial cells	11
Fibroblasts	5
Macrophages A	21
Macrophages B	19
Mast cells	14
mDCs A	12
mDCs B	33
Monocytes	18
pDCs	13
RBCs	15
T cells & NK cells	40
Tuft cells	32

Table 4: Detailed cell type information of the scRNA-seq dataset GSE111672 (PDAC-A).

Cell type	#
All	1733
Acinar cells	6
Cancer clone A	339
Ductal - CRISP3 high/centroacinar like	152
Ductal - MHC Class II	211
Ductal - terminal ductal like	736
Endocrine cells	13
Endothelial cells	159
Macrophages	9
Mast cells	13
mDCs	35
Monocytes	20
RBCs	3
Tuft cells	37

Table 5: Detailed cell type information of the scRNA-seq dataset GSE111672 (PDAC-B).
